# The OCR-Vx experience: lessons learned from designing and implementing a task-based runtime system

**DOI:** 10.1007/s11227-022-04355-0

**Published:** 2022-03-02

**Authors:** Jiri Dokulil, Siegfried Benkner

**Affiliations:** Faculty of Computer Science, University of Vienna, Vienna, Austria

**Keywords:** Parallel computing, Task-based runtime systems, Open Community Runtime, Non-uniform memory access

## Abstract

Task-based runtime systems are an important branch of parallel programming research, since tasks decouple computation from the compute units, giving the runtime systems greater flexibility than a thread-based solution. This makes it easier to deal with the ever-increasing complexity of parallel architectures by providing a separation of concerns—the specification of parallelism is separated from the implementation of the parallel computations on a specific architecture. The Open Community Runtime is one such system, aimed at large-scale parallel systems. Unlike many other task-based runtime systems, the creators not only provided an implementation but there is also a comprehensive specification document. This has allowed us to create an independent implementation, called OCR-Vx. In this article, we present our experience of developing the runtime system, put our work in the context of the specification and the other implementations, and describe key lessons that we have learned during our work. We discuss the design and implementation issues of task-based runtime systems and applications including task synchronization and scheduling, data management, memory consistency, the relation between shared-memory and distributed-memory runtime systems, NUMA architectures, and heterogeneous systems. The article is aimed at audiences not familiar with OCR, since we believe these lessons could be valuable for developers working on other task-based runtime systems or designing new ones.

## Introduction

The Open Community Runtime (OCR, [37, 38]) is an open specification of a distributed task-based runtime system for extreme-scale parallel systems. It uses parallel tasks to express computation parallelism and decouple computation from compute resources. Task-based runtime systems are considered to be a promising way for addressing the challenges of programming future parallel systems, since they have greater control over the execution of the application. This allows the runtime systems to more easily adapt a task-based application to dynamically changing execution conditions, like performance variability in both hardware and software.

The task-based design of OCR is similar to other task-based runtime systems like StarPU [3], TBB [31], or HPX [29]. However, there are some differences in how exactly these tasks are defined and synchronized. In particular, OCR uses a task-like approach to deal with data as well. All non-transient data in an OCR program are stored in data blocks managed by the runtime system. This decouples the data from storage in a similar way that tasks decouple computation from compute units.

Together, tasks and data blocks in OCR provide a very “pure” compute environment, since there is no computation outside of tasks and no data outside of data blocks, apart from local variables of currently running tasks. The stand-alone specification document [37] provides a clear definition of how an OCR runtime should behave and the OCR API defined in the specification is very compact, with only a small number of functions with clearly defined responsibilities. Together, all these attributes make OCR a very interesting option for research.

Since 2014, we have been developing an implementation of the OCR specification called OCR-Vx [18, 24]. We have targeted both shared-memory and distributed-memory parallel architectures, as well as some less common architectures like the Intel Xeon Phi (both in the KNC coprocessor [18] and KNL processor format [19]) and OpenCL accelerators. We have also made multiple contributions to the OCR specification and developed a detailed consistency model for OCR [15]. A lot of our later work focused on the way OCR can be efficiently executed on non-uniform memory architecture (NUMA) systems [20], since we have discovered that the aspects of the OCR design originally aimed at distributed memory systems open up some interesting possibilities also for shared-memory NUMA systems.

This article summarizes our work and puts it into the wider context of parallel runtime systems. Although some aspects have already been published [15, 17–20, 23, 24], the purpose of this article is to provide the big picture that is hard to see from the individual papers and to discuss interesting topics that are relevant to a wider audience but have not yet been presented elsewhere. We explain why certain design decisions made during the creation of OCR and OCR-Vx are more significant than they might initially appear and how they impact the runtime system implementation and/or application programmers. The paper is aimed at people familiar with task-based runtime systems, but does not require detailed knowledge of OCR as the fundamental concepts of OCR are provided in the paper. Our goal is to convey the lessons learned from our work on OCR-Vx, without burdening the reader with details about OCR. We believe this information could be beneficial to anyone working on another existing task-based runtime system or designing a new system.

The specific contributions and topics covered by this article include a discussion of the effect a formal specification document has on the whole ecosystem, the design trade-offs in a distributed runtime system, the OCR consistency model, novel approaches to mapping SPMD applications to task-based applications, the reasons for having three different OCR implementations, the NUMA support in our OCR-Vx implementation, and experimental performance results with different benchmarks on a variety of different architectures.

The rest of this paper is organized as follows. First, Sect. 2 introduces the key concepts of OCR and discusses trade-offs made by its designers. Section 3 discusses the importance of the OCR specification and what role it played in the development of OCR-Vx. We also explain some important extensions that have been added to the specification later. Our implementation, OCR-Vx, is introduced in Sect. 4, providing a comparison of the three different OCR implementations that are part of the OCR-Vx suite. The support for heterogeneous and NUMA architectures is also explained in this section. Section 5 covers some of the important applications built on top of OCR, how they perform in OCR-Vx and how that performance relates to alternative systems (like OpenMP). Related work is provided in Sect. 6. Section 7 concludes the article.

## The Open Community Runtime

The Open Community Runtime is an asynchronous many-task runtime system. The original development started as part of the US Department of Energy XStack project. The OCR was a software component of the Traleika Glacier (TG) HW/SW co-design project [25], which also developed the Traleika Glacier hardware architecture [13]. The main motivation for the creation of OCR was to provide a low-level runtime system, which could be used to build a wide variety of high-level programming models using a very limited set of core concepts. Application developers would not use OCR directly, targeting the high-level models instead. Because the TG hardware was built not just around heterogeneous compute cores, but also with an innovative memory architecture (with hierarchical scratch-pad memory and global address space), the OCR not only uses asynchronous tasks to manage massive parallelism, but also relocatable data blocks to manage application data. Although there are other runtime systems that can also manage application data (like Legion [6] or StarPU [3]), there are significant differences in the way OCR data blocks work (see related work discussion in Sect. 6). The OCR was also designed to provide fault tolerance, which is an important concern for extreme-scale architectures, and data blocks play an important role in facilitating resilience.

### OCR basics

The following section introduces the most basic OCR concepts. Readers familiar with OCR may continue directly to Sect. 2.2.

Tasks and events In OCR, all computation is performed by tasks. Task themselves are serial and parallelism is achieved by running multiple tasks concurrently. The OCR tasks are sometimes called *event-driven tasks* (EDTs), because they are synchronized by events. OCR tasks are non-blocking—once an OCR task starts, it should run to completion without blocking. All synchronization needs to be performed before the task starts, using events. Events are lightweight objects whose main purpose is to define when tasks are allowed to start. The most basic example is a dependency between two tasks, where task A needs to finish before task B can start. In OCR, this is accomplished by connecting the output of task A to an event and then using that event as an input of task B. This connection can also be used to pass data from task A to task B. In an actual OCR code, there is no need to create a new event to build this simple connection, since whenever a task is created, an *output event* is created automatically for this task. This event represents the completion of the task. In our example, this event would be used as the input for task B.

There are other, more complex types of events, but the basic idea is the same—to define when a certain task is allowed to start and potentially also pass data from one task to another. The actual time and place where the task is executed are determined by the scheduler of the runtime system. Since all work of an OCR application is performed by tasks, this also includes the creation and management of tasks and events. There is no “main thread” that would run the program and spawn tasks when some parallel computation is necessary, which is the usual architecture used by OpenMP, TBB, StarPU, and many others.

Data blocks To be able to utilize the TG architecture (e.g., the scratch-pads), the runtime system needs not only the ability to move computation (tasks) around, but also the data. Therefore, OCR requires all application data to be stored in data blocks, which are controlled by the runtime system. The applications are responsible for mapping their data to data blocks. When a task is running, it can use local variables on the stack in the usual way, but any data that need to persist outside of a single task have to be stored in an OCR-managed data block. From the runtime system’s point of view, a data block is just a contiguous block of memory (a sequence of bytes) with no internal structure or meaning. The data block is guaranteed not to move while a task is using it, but the runtime system can move it around in memory otherwise. It can also (in a distributed environment) move it to another node or even make multiple copies of the data block. To facilitate this, each task needs to declare a set of data blocks it will be using and also the kind of access (read-only or read-write, with different coherency options) it needs. The runtime system uses this information to decide where to place the data, but it also influences task scheduling. For example, it is not possible to concurrently run two tasks that require exclusive write access to the same data block.

One interesting consequence of the way tasks request data blocks is that data movement in OCR is handled in a declarative way, rather than imperatively. The OCR application declares that a certain task needs access to some data block and it is up to the runtime system to ensure that the data are moved or copied to the appropriate location. This is different to message passing or PGAS systems, where the transfers are initiated explicitly by the application code. Once again, the idea is to move control from the application to the runtime system [37]. In some situations, the runtime system might be able to achieve a zero-copy behavior, by rearranging tasks instead of transferring the data.

**Fig. 1:**
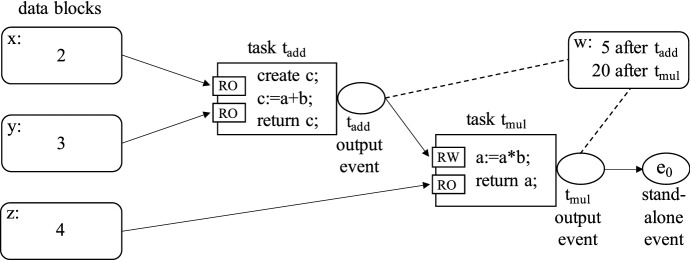
Example of an OCR program that computes $(x+y)*z$. A detailed explanation is in the text below

For an example of a simple OCR program, see Fig. 1. It demonstrates how to use two tasks to compute $(x+y)*z$ on data stored in multiple data blocks. Each of the three inputs is stored in a separate data block. The work is split into two tasks, with $t_\mathrm{{add}}$ doing the addition and $t_\mathrm{{mul}}$ doing the multiplication. The RO and RW annotation on the task inputs determines if the data block is provided with read-only or read-write access. Inside a task, the data blocks are referred to using local names *a* and *b*, not their global identifiers. The $t_\mathrm{{add}}$ performs $x+y$, because *x* is passed as the first argument *a* and *y* is passed as the second argument *b*. The task creates a new data block *c* (with global identifier *w*), stores the result of $a+b$ in *c* and returns *c*. The block *w* (which used to be *c* in $t_\mathrm{{add}}$) is then passed from the task’s output event to the first input of the $t_\mathrm{{mul}}$ task, where it is then available as *a*. The $t_\mathrm{{mul}}$ task modifies the contents of the *w* data block (it has read-write access) to contain the result of the multiplication and then returns the block. The data block is then sent via the task’s output event to another event $e_o$, which can be further connected via dependencies to other tasks that need the $(x+y)*z$ value. At this point, no other task knows the identifier of block *w*, so the result stored in *w* can only be obtained by setting up a dependency on $e_o$. Please note that due to task execution overhead, tasks in a real OCR application should do a non-trivial amount of work, so this organization would only make sense if the values were not scalar (e.g., vectors or matrices).

Hints Another important OCR concept is *hints*. Hints are additional information passed from the OCR application to the OCR runtime system, which convey application knowledge related to execution performance to the runtime system. In a distributed environment, this could mean task and data block affinities, suggesting the best placement for the tasks and data blocks. Another example of a hint is a flag indicating that a certain data block should be placed in high-bandwidth memory on architectures where it is available.

GUIDs Finally, one feature of OCR that we need to point out is the globally unique identifiers (GUIDs). All OCR objects (tasks, data blocks, events, but also other helper objects) are referenced using these identifiers. These identifiers form a global name space and they allow a task to use any object irrespective of where the object is actually placed in a distributed environment. The task only needs to provide the GUID and the runtime system is responsible for locating the object and performing the action on the (possibly) remote node.

In the existing implementations, GUIDs are 64 or 128 bit objects opaque to the application. Internally, they may encode information about the type of the object or the location of the object (in a distributed environment). In our shared memory OCR implementation, a 64 bit GUID is actually a pointer to an OCR object, which is also a valid option, since GUIDs are allowed to be recycled.

### Design trade-offs

OCR, just like any other such system, needs to make certain trade-offs. In the following section, we will discuss the benefits and drawbacks of requiring all computation to be done by tasks and all data to be stored in data blocks, benefits of making tasks non-blocking, and the importance of API size.

In general, giving more control to the runtime system often requires constraining application developers that use the runtime system. On the other hand, it allows the runtime system to be more efficient or it may simplify the application developers’ job. For example, in OpenMP, a parallel for loop requires the number of iterations to be known before the for loop is entered, restricting what kind of for loops can be parallelized using OpenMP. But it allows OpenMP to run the for loops efficiently at a degree that would not be possible if the termination condition (and the number of iterations) could not be evaluated beforehand.

**Fig. 2:**
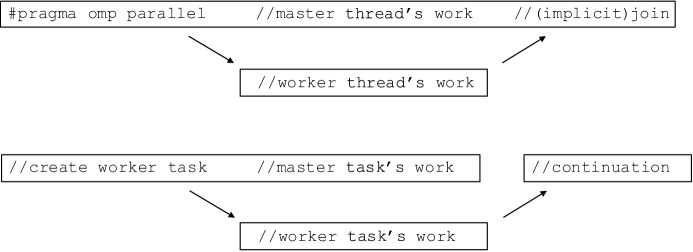
Comparison of OpenMP fork-join (top) and the OCR mapping of the same pattern (bottom). The boxes represent OpenMP threads and OCR tasks. For simplicity, the events used to synchronize OCR tasks are left out. The OpenMP master thread needs to be split into two tasks to facilitate synchronization with the worker; they cannot be mapped 1-to-1

Task-only environment One such restriction imposed by OCR is the aforementioned requirement to run everything as a task. To map a thread-based model like the basic OpenMP fork-join model to OCR, one might consider simply converting threads to tasks in a 1-to-1 fashion. This covers the “fork” part of the fork-join model used by OpenMP, but due to restrictions placed on OCR tasks, it is not possible for one OCR task to wait for completion of another OCR task. This complicates the “join” operation, where the master thread waits for the other threads in the team to finish. Such synchronization can only be achieved through OCR events. For example, to join two threads represented by tasks A and B, a task C needs to be created and dependencies (via events) set up so that C runs after both A and B finish. An overview is shown in Fig. 2. Splitting the master thread into two tasks might look like a minor technical detail, but it is made much more significant by the fact that it is only possible to pass information between tasks using data blocks. It is not possible to make local variables of tasks A or B available to task C without storing them in a data block and making that data block accessible from C. This demonstrates some of the issues in mapping other programming models to OCR.

**Fig. 3:**
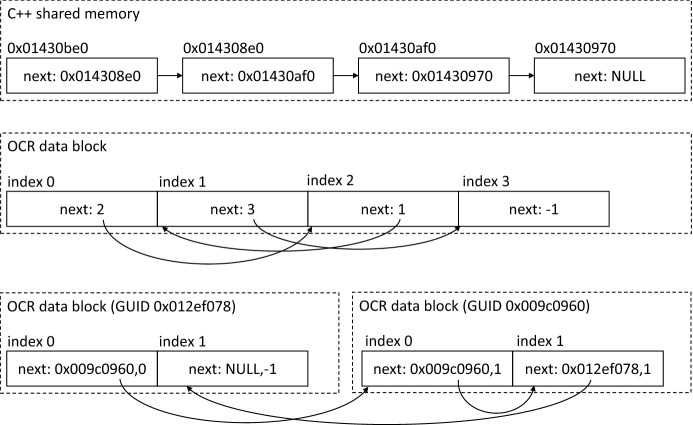
Comparison of a linked list with 4 items representation in C++ using pointers to the next item (top), OCR using indexes within a single data block (middle), and OCR using the pair of a GUID and an index to point to the next item (bottom). For illustrative purposes, we assume that both pointers and GUIDs are 32 bits long

Complex data structures The ability to move data blocks is very useful for the runtime system, especially in a distributed environment, where it facilitates all data transfers between tasks running on different nodes. It can also allow the runtime system to use specialized (e.g., high-bandwidth) memories or scratchpads. But it requires special care to be taken when dealing with complex data structures. For example, these data structures may be serialized when they are stored in data blocks. However, serialization is not the only option, it is still possible to use complex data structures in OCR data blocks.

However, one important restriction imposed by the use of data blocks is that it is not possible to use pointers in these data structures. Any pointer to data in a data block is only valid within one task. Another task (even if it is running at the same time) might get access to the same data block via a different base pointer (pointer to the start of the data block). To persistently reference a place in a data block, instead of a pointer one needs to identify the data block and an offset inside the data block. The data block identification could be implicit, like in the case of all offsets being local to the same data block. The most general way of representing a pointer to data in a data block is a pair of GUID of the data block and an offset inside the data block. An example that compares the different variants is shown in Fig. 3. For application developers, there would be some minor technical difficulties of using the larger (64/128 bit GUID plus 64 bit offset) compound identifiers, but they are comparable to C++ iterators and a similar streamlined C++ interface could be created (if C++ is used; OCR can support C applications).

However, distributing a single data structure across multiple data blocks raises another problem. Remember that every task needs to declare the data blocks it wants to access before it is started. So, if a task needs to access an OCR “pointer,” but the GUID part identifies a data block that the task does not have access to, there is no way for the task to access the data. It is necessary to create a new task and give it access to that data block. There are two basic ways of dealing with this situation. First, the application might always create a list of all data blocks that a task will need and request access to these data blocks. This could be trivial for applications with regular data access, but create significant overhead for applications that use pointers to access data from a large set of data blocks. The second alternative is to write the application so that if a task encounters a point where access to an unavailable data block is required, the state of the computation is saved and another *continuation* task is created to continue with the computation but this time having the access to the data block. The MiniAMR [45] OCR application is written this way, but the cost in terms of code complexity is quite high. Keep in mind that the continuation task could be moved to a different node in a distributed system and that the data blocks may have been moved (invalidating any pointer stored in the computation state information) or modified by other tasks in the period between the end of the original task and the start of the continuation.

Data movement It may appear that data blocks are the biggest stumbling block for OCR application developers. Based on our experience, this is actually the case. Deciding how and where to store the data and then ensuring that it is available to the tasks that need it can be tricky. But the data blocks are also one of the most interesting features of OCR. In programming models based on the message passing interface (MPI) or partitioned global address space (PGAS) models, communication is explicit and it is up to the application to figure out what data to send where (and when). In OCR, a task makes data available by storing it in a data block and then another task consumes it by letting the runtime system know that it needs the data block as one of its inputs. The runtime system might move the data block to the node where the consumer task is going to be executed, create a copy of the data block on that node, or move the task to wherever the data block already is. This declarative approach is used by many high-level systems (e.g., Map-Reduce), but it is less usual for a low-level architecture like the OCR.

Deferred execution model Another important consequence of the way data blocks are used in OCR is the deferred execution model, which allows the runtime system to delay the effects of operations performed by a task, if necessary. This allows the runtime system to improve performance of running tasks and it can also help with implementing resilience in the runtime system (see Sect. 4.2.4 for a more detailed discussion). But first, we need to revisit the problem of accessing data blocks that are not known in advance when the task is being set up.

A different (and rather obvious) idea for solving the problem is to allow tasks to acquire additional data blocks while they are already running. This has actually been investigated and it is available in the OCR specification via the “legacy” API. However, it is not considered to be a good solution. Acquiring new resources while already holding other resources might lead to deadlock, but the actual argument against this solution is a different one. It goes against an important part of the OCR design philosophy. Once an OCR task starts, it should be able to proceed with its execution irrespective of what the other parts of the system are doing (non-blocking tasks). It should not stop and wait for a data block to become available. In fact, it should be possible to go even further and use a *deferred execution model*, where the task runs but the changes it makes to the global state of the execution do not have an immediate effect. While this is not directly at odds with giving tasks the ability to acquire new data blocks during execution, it could significantly increase the time such tasks need to wait, since they may be waiting for results whose effect was deferred. The OCR design philosophy is to let the runtime system do most of its work before the task starts and then let the task run without interruption.

API size Another part of OCR design philosophy is to keep the API small. From a research point of view, this turned out to be very useful. There are only around 20 functions in the core OCR API (plus half of that in the extensions) and even then a significant portion of those are minor helper functions (e.g., comparing GUIDs). Once an application finishes setting up^1^, it could work with as little as two functions (create task, set up a dependency) if it does not need to create any more data blocks. To create and destroy new data blocks, two more functions are needed. So, when we needed to study the impact of some change on the whole OCR API, it was a fairly simple job. On the other hand, having a very small choice of synchronization options made it difficult to map certain synchronization patterns to OCR. There are only four types of events in OCR and three of them have very similar behavior, differing only in the way their lifespan is managed. This brings down the number of actual options to two. While it was still possible to map most applications to OCR, it was surprisingly difficult to efficiently replicate the behavior of regular iterative single-program multiple-data (SPMD) codes, e.g., MPI. Adding *channel* events (for a detailed discussion of channel events see Sect. 3.2) had a very small impact on the OCR API and it was not too difficult to implement, but it had a huge effect on these codes, making them not only much simpler, but often also more efficient. On the other hand, there were proposals for other kinds of events, but most of them would only benefit very specific applications and they were not adopted.

Overall, the OCR presents an interesting set of design decisions, many of which were motivated by the properties of envisioned future architectures (like the TG), but they make OCR interesting for research into runtime systems. OCR has a small and clean API and it gives a very strong role to the runtime system. The downside of this novel and small API is the difficulty of porting existing applications to OCR. But if application data are regular (to avoid the “unknown GUID” problem), writing a new application is not that challenging for someone familiar with a similar task-based runtime system, like the TBB.

## Impact and evolution of the OCR specification

One significant difference between OCR and many similar projects was the existence of a formal specification [37]. Early in the project, the authors have decided to write a standalone specification document that would define the OCR API and the expected implementation behavior independently from the actual implementation. At the time we got involved with OCR, the specification was at version 0.9, which was the first public release of the document. At this point, most of the design and the API had been finalized and the subsequent versions were mostly minor improvements and changes. Since version 1.1.0, we became directly involved in the specification, making direct contributions to the document.

Implementations There are multiple implementations of the OCR specification. The original implementation created within the X-Stack project along the specification is usually referred to as XSOCR (X-Stack OCR, [38]). Based on this implementation, P-OCR (Performance-OCR, [32]) was created at the Pacific Northwest National Laboratory. Our implementation is the Vienna OCR (OCR-Vx, [23]). Actually, OCR-Vx is a collection of three different implementations, which are described later in Sect. 4.

The existence of the OCR specification was greatly beneficial for our work. We were able to take the specification (version 0.9) and create a completely independent implementation of the OCR. The only common parts of XSOCR and OCR-Vx are the API header files. While these could easily be re-created for OCR-Vx, it was not necessary. The header files only declare the functions exposed in the OCR API and define the various constants. In fact, it is even beneficial to have identical headers, as it allows for library interchangeability. It would be possible to compile an OCR application and choose the runtime to use at link time, by linking either to XSOCR or one of the OCR-Vx implementations. We have actually managed to successfully test this^2^.

Implementation interoperability While interoperability among different runtime implementations was good, there were cases of applications developed using one OCR implementation that did not work out-of-the-box with another one. While some of these were due to bugs in the applications or the runtime systems, there were multiple cases where we discovered that the specification was not clear enough and different runtime system implementations had different views on what the expected behavior should be. To give one example, when creating an event, it is possible to specify that the event can be used to pass a data block along the dependency chain that it is a part of. It is possible for an application to not specify this flag and still try to pass a data block through the dependency. One runtime system implementation considered this to be an error, while a different implementation interpreted this to mean that the data block should be ignored. The specification was updated to clarify the required behavior.

Deferred execution model Another subtle (but important) change made to the OCR specification influenced by our work was to support the aforementioned deferred execution model. In this model, the changes to the global application state made by a task are not required to be made immediately, facilitating uninterrupted execution of tasks in a distributed environment. The OCR API required certain information returned from API calls, mostly error codes for bad API calls. However, in a deferred execution model, the information required to identify such errors is not necessarily available immediately. For example, consider two tasks that attempt to delete the same object (an illegal operation in OCR) and the delete operation is deferred to a later point in time. The error code requirements were relaxed by later specifications for this reason. Overall, the specification now follows the idea that a task should not be able to query the state of the various OCR objects, as this violates the basic OCR principle that once a task starts, it should run to completion without interruptions and delays. Dependencies are the OCR way to ensure that the computation has reached a certain state.

Impact on application development Just like tasks decouple work from compute units and data blocks decouple data from storage, the specification allowed a certain level of decoupling between the people developing the runtime and the people working on the applications, even though they were mostly from one team working in the same building. It greatly helped in preventing the addition of features to the runtime that would only serve one application. While it is easy to just skip across the source code boundary from the application into the runtime system and make a few changes, it is much harder to do where there is a specification for the interface, which would have to be updated as well. Changing the specification would automatically involve more people in the process and the specification follows a much slower release schedule.

Such requests would occasionally emerge, but in many cases, it turned out that the change was not necessary and the desired functionality could be achieved with creative use of existing features. On the other hand, there were cases where even a fairly notable change had to be made to the API, based on the needs of application developers. The most notable example is the addition of the aforementioned channel events. Since they are quite an interesting concept with a surprisingly big impact, we will explain them in greater detail in Sect. 3.2.

### Memory model

Since the first released version, the OCR specification provided a memory model. It was built around the happens-before relation, similar to the Java and C++ memory models [8, 36]. In OCR, the relation is defined by the way tasks and events are connected with dependencies. The happens-before relation is then used to define the visibility of changes made by tasks to data in data blocks.

The memory model only deals with the data inside of data blocks, not OCR objects (tasks, events, data blocks, etc.) as such. So, if two tasks modify the state of some runtime object, it is not clearly defined how are these changes applied. The expected behavior is usually clear from the context and implicit assumptions one makes about the programming model, but they are not explicitly present in the specification. OCR application developers tend to assume that these changes are instantaneous, but this is not necessarily true in a distributed environment or when the deferred execution model is used. In practice, this view is usually good enough, but there are situations, where they fail. This failure usually surfaces as a race condition and an application crash. Without a well-defined model, it may be difficult to find the problem and impossible to decide if it is a bug in the application or in the runtime system.

We were able to extend the OCR memory model [15] to cover all OCR objects. There is now a clear definition of the order in which the operations on these objects should happen, based on the happens-before relation. We can also see if there is no such ordering between two operations, creating a race condition. While building the memory model, we also filled in some missing pieces in the way happens-before was defined [15] . All OCR-Vx implementations as well as XSOCR implementation adhere to this extended memory model. The XSOCR required no changes. With OCR-Vx, the situation was more complicated. Early OCR-Vx implementations did not strictly follow the model (leading to race conditions and crashes), before we redesigned the relevant pieces to adhere to the model. However, it was the creation of this new version that actually inspired the creation of the memory model, not the other way round. We have created the memory model to confirm that the new implementation was correct. Section 4.2.2 discusses this in greater detail.

### Channel events and SPMD

Channel events are a special kind of events added to later OCR specifications by the XSOCR team. They allow a single event to be used multiple times, to pass multiple data blocks, thus forming a channel between different parts of the application. The introduction of channel events required a minor extension of the specification, a slightly more significant (but still very limited) change in the implementations, but provided a major benefit for multiple applications. The channel events also proved to be a bit of a challenge for the OCR consistency model, but it was still possible to fully integrate them.

The original motivation for channel events came from a larger effort to find a good way to map SPMD applications based on message passing to OCR. In the SPMD model, there are independent processes that communicate by sending messages between the processes. We have already discussed the problem of mapping multi-threaded applications to tasks. Similar approaches can be used to map processes to tasks. However, it is not so easy to map message passing to tasks and data blocks.

A common pattern for high-performance applications is to repeat a large number of iterations, often as a result of temporal discretization (time-marching methods). Due to data dependencies, most parallelism is found inside individual iterations. As a result, in a many-task model like OCR, each iteration is represented by a large number of tasks. Due to the high number of tasks in each iteration and the high number of iterations, it is not possible to create all of the tasks upfront, as the memory overhead would be too large. In a massively parallel environment, it would also not be practical to have a single thread or task create all tasks for a single iteration, since it would create a bottleneck. As there is often a 1-to-1 mapping of tasks between subsequent iterations, the common solution is to have every task in one iteration also create its counterpart in the subsequent iteration. In reality, the pattern could be more complicated than 1-to-1, but the general idea is the same as far as the channel events and their use is concerned, so we will assume the 1-to-1 pattern for simplicity. Consider the following code: 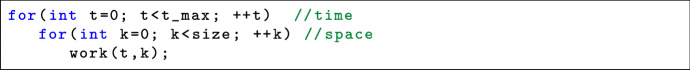


The outer for loop represents the iterations over time, the inner for loop (e.g., spatial discretization) provides the parallelism, and work(t,k) is the task to run. In a typical OCR implementation, the work(t,k) task would also create the work(t+1,k) task. This is fairly trivial to achieve. The problems arise from the fact that the tasks also consume and produce data. Passing a data block from work(t,k) to work(t+1,k) is also easy, since the latter is created by the former. The work(t,k) task knows the GUID of the work(t+1,k) task because it is returned by the OCR API call that is used to create the task. With this information, a data block can be passed using a single OCR API call.

However, it is also quite possible that work(t,k) needs to send data to work(t+1,k+1). With MPI, the application could be implemented by assigning different values of k to different processes (where k is equivalent to the rank of the process) and having each process perform all iterations of the outer loop for that k. The aforementioned data exchange would then be equivalent to sending a message from the process with rank k to the process with rank k+1. Since work(t,k) does not know the GUID of work(t+1,k+1), it cannot pass a data block to it directly. It is possible to use an event connected (by a dependency) to the task work(t+1,k+1) and use the event to forward the data block to the task. However, just like tasks, simple events in OCR are single-use. Once a task finishes running, it is automatically destroyed. Similarly, once an event serves its role of propagating a signal (optionally accompanied by a data block), it is also destroyed. There are different kinds of events with different lifespans, but they are also single-use as far as passing a data block is concerned. Therefore, the problem of not knowing the GUID of the recipient task would only be transformed into a problem of not knowing the GUID of the event (if the event is created by work(t,k)) or not being able to connect the event to the task (if the event is created by work(t,k+1)).

**Fig. 4:**
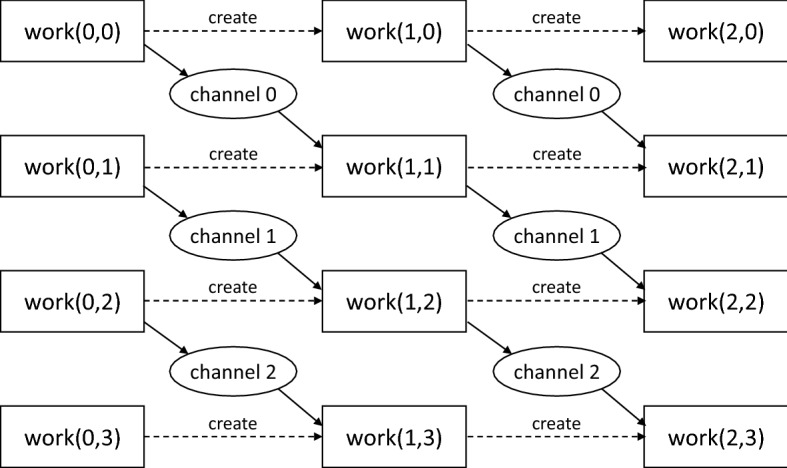
Visual representation of tasks that implement the first three iterations of an application that uses channel events to communicate between tasks with different creators. Note that each pair of shapes labeled “channel k” represent the same channel event being used multiple times to send a data block from work(t,k) to work(t+1,k+1)

Channel events change this, since channel events can be used multiple times to pass a data block. So, we can create k channel events at the beginning and make all of their GUIDs know to all work tasks, across all iterations. This is easy to achieve when done at the beginning. When work(t,k) wants to send a data block to work(t+1,k+1), it sends the data block to the k-th channel event. When creating work(t+1,k+1), work(t,k+1) connects the channel event to an input of work(t+1,k+1). This way, we have a path from work(t,k) to work(t+1,k+1). An example is shown in Fig. 4. It is possible to work around the problem and not use channel events, but all the alternative solutions used in the existing OCR applications significantly increase the code complexity and/or add runtime overhead.

*Acknowledgment* At this point, it is important to clarify that channel events are *not* our contribution. They were designed by the XSOCR team. We include the detailed discussion here, since the channel events are an important concept that fundamentally affects OCR, but as far as we know, they have not been described in any publication by their designers.

Due to their re-usable nature, channel events are tricky to deal with in the consistency model, but there was a way to add them to the model in a consistent manner. The basic idea is to define how the happens-before relation among operations on the source side and the happens-before relation among operations on the destination side affects the flow of the data blocks. If we know that putting data block A into a channel event happens-before putting data block B into the channel event and a request that task X should get an output from the channel event happens-before a request that task Y should get an output from the channel event, the consistency model guarantees that X gets A and Y gets B. We consider the fact that channel events could be added to the consistency without changing their intended semantics and with no changes to the basis of the model a confirmation of a good design of both.

In parallel to the development of channel events, we have worked on an SPMD library for OCR. The library used a data block and two types of tasks (push and pop) to build a message queue that could be accessed from different tasks without the need to directly synchronize these tasks. It can provide behavior that is equivalent to channel events. This is interesting, as it shows that channel events do not fundamentally extend the OCR programming model. However, they are easier to use and more efficient, making them a good addition to OCR.

With channel events, a lot of complexity is removed when mapping SPMD codes to OCR. But it is still necessary to break up the processes into tasks. For MPI codes, the channel events only replace point-to-point communication. There is a special kind of events (latch events) which can be used to build a distributed barrier. But the other, more complex MPI collective operations have no direct counterpart in OCR. The XSOCR team has developed a library for efficient reduction operations and something similar would be possible for the other operations. However, it would still be a challenge to actually match the efficiency and convenience of MPI.

### Labeled GUIDs

Labeled GUIDs are an extension to the OCR specification allowing users to create a range object, which represents a collection of OCR objects accessible via their indexes in the range. So, given a GUID of the range, it is possible to make an API call to get the GUID of the third object in the collection. Initially, these GUIDs do not correspond to actual objects, but an object can be created using that GUID and then the GUID will identify that object. This was originally intended as a solution for the scenario described in the previous section about channel events.

The work(t,k) tasks could be stored in a map, indexed by k. However, there is an issue with object lifetime, which was not fully addressed by the specification. To use labeled GUIDs to solve the problem, we need to use the same range for all iterations, so when work(t,k) is running, we need to ensure that the range points to work(t+1,k+1). This is required in order to allow work(t,k) to pass data to work(t+1,k+1). In our proposal, work(t,k+1) is removed from the map when it starts. Furthermore, it creates work(t+1,k+1). Combined, work(t,k+1) needs to start and run to completion to ensure that the map is in the correct state for work(t,k) to be able to pass data to work(t+1,k+1). Having such dependence from work(t,k+1) to work(t,k) is not practical.

Using multiple tasks, this situation can be resolved, but it increases complexity and overhead of the application. The channel events have turned out to be a much better solution for this scenario, effectively stopping the development of labeled GUIDs.

### Local identifiers and I/O

As part of our work on OCR, we have also created several extensions to the specification [17]. Local identifiers were an attempt at solving the aforementioned lifetime problem of labeled GUIDs, but channel events made them also obsolete, solving the problem with only very limited changes to OCR. The same is true for another proposed extension, which attempted to solve another labeled GUID issue, regarding the responsibility for object creation.

The other two extensions proposed by us dealt with data blocks. The first one was the introduction of file-backed data blocks, providing basic file IO in a way consistent with the OCR design principles. The idea is very similar to memory-mapped files provided by most modern operating systems. A memory-mapped file could actually be used to implement a file-backed data block. Mapping the whole file into one data block may be too coarse. While it would be possible to only map a part of the file, we have proposed an alternative solution, which allows any data block (not just file-backed) to be partitioned into a number of small non-overlapping chunks, each of which behaves as an independent data block. This has the benefit of allowing tasks to access parts of a data block with different access modes, for example specifying exclusive write on the first half, but just read-only on the second half, allowing other tasks to access the second half concurrently.

As none of the OCR applications that we are aware of have reached the state where a file input and output on a large scale would be required, the extensions were never adopted by the applications and were only available in some version of our OCR implementation.

## OCR-Vx

In the following section, we will discuss our OCR implementations. There are three separate OCR implementations in OCR-Vx. OCR-Vsm is a shared-memory parallel implementation, OCR-Vdm is a distributed memory parallel implementation, and OCR-V1 is a shared-memory serial implementation. These were not created at once, but were added and extended over several years. We will discuss each of them, but first, let us start with a brief history of the project, to put the development into context.

### Brief history of OCR-Vx

Before getting involved with the OCR, we worked with the early Xeon Phi coprocessors, developing a heterogeneous task-based runtime [16]. It exposed the offload programming model of the coprocessors through parallel algorithms, inspired by the way that TBB parallel algorithms expose multi-core parallelism. The OCR was an interesting possibility to take this even further and hide the offload model already on the task level, since OCR tasks and data blocks could be made to freely migrate between the host and the co-processor. We started by creating a new implementation of the OCR specification, targeted specifically on the Xeon Phi coprocessors [18].

Like in our previous work, we have used the TBB task scheduler in our implementation, since the highly tuned task-stealing scheduler fit our purposes very well and we had a good experience with it on the Xeon Phi. The TBB scheduler is based on task-stealing (work-stealing). We have used the same approach also in all of the later, custom schedulers designed specifically for OCR-Vsm.

Note that this OCR implementation was not using the heterogeneous host/coprocessor setup. It only executed tasks on the coprocessor. Before the heterogeneous variant, we decided to implement a non-heterogeneous distributed OCR runtime system that would run on traditional MPI clusters. In the end, since the development of the Xeon Phi coprocessors ceased soon after, we never actually implemented the heterogeneous OCR for them.

At this point, we had an implementation of OCR for the Xeon Phi coprocessors. Since the coprocessors mostly behave like normal x86-64 machines, it only required some tweaks to turn that implementation into a more general OCR implementation for shared memory systems. This would end up being called OCR-Vsm.

The single-threaded OCR-V1 implementation was actually the last addition to OCR-Vx. Difficulties that the application developers had working on OCR applications and our work in the OCR memory model motivated the creation of a special OCR implementation, which sacrificed performance in favor of significantly improved error detection and diagnostic features.

### OCR-Vdm

Our distributed implementation of OCR [24] is called OCR-Vdm, becoming the second part of our OCR-Vx suite of OCR implementations. It can use either sockets or MPI for communication. The GUIDs encode the node where the object is stored and an index into the object lookup table on the node. Each object is stored on one node (the owner), but it is possible to store copies of a data block on nodes other than the owner. Similar to the XSOCR at the time, there was no distributed task scheduler. OCR-Vdm placed each task to a node when it was created and it would not migrate to other nodes. By default, a task would be placed locally on a node that executed the task that created it, although there was a (very inefficient) round-robin option that would spread the tasks across all nodes. To place a task on a remote node, the application would have to provide a hint when creating the task explicitly dictating the placement of the task. The OCR-Vdm runtime system only automatically schedules tasks to cores within a single node, but not between nodes (except using the user-specified hints).

#### OCR-Vdm single process pseudo-distributed environment

Apart from the aforementioned MPI- and socket-based distributed implementations, OCR-Vdm had another variant, which proved to be very useful for development and debugging. It was possible to emulate distributed execution on an *N*-node cluster inside a single process on one machine. All runtime components were replicated *N* times. Each task was aware to which replica it belongs and used that to access the runtime system. To communicate, the replicas used the normal OCR-Vdm communication interface, but instead of MPI or sockets, it used the fact that all replicas share the same memory space to exchange data.

This setup made it possible to debug most of the distributed runtime system and applications with just a single process, giving one local debugger instance full direct access to the whole runtime system and application state. The communication subsystem is a very small, isolated component and everything else can be debugged like this. This has proven to be extremely useful on multiple occasions, but the most prominent was debugging of the way the distributed runtime system manages distributed copies of data blocks. The ability to stop all of the “nodes” at exactly the same time (e.g., through a conditional breakpoint) and explore their state in a debugger session made this process much easier. Afterward, no further bugs were discovered in this part of the runtime system even in a truly distributed environment.

#### Distributed state management

OCR-Vdm needs to maintain a distributed state of the OCR application and the runtime system. This also includes objects like tasks, events, and data blocks. The state of these objects is changed either by the application through the OCR API or by the runtime system itself in response to certain events. Some of these changes are simple, only changing the state of one object. However, certain operations modify the state of multiple objects. An example is adding a dependency that connects an event to a task. This needs to modify the event making it aware of the fact that a task now depends on it. But it also needs to tell the task that one of its inputs is coming from that event, because the operation also defines the kind of access the task will get to a data block received from the event.

Now, let us assume that the dependency was added by a task running on node N, the event is on node E, and the task is on node T, with those being three distinct nodes. We could just send messages to both T and E, asking them to make the required changes. Let us call the messages *m*1 and *m*2. Most of the time, this would (and did) work fine. However, there is a possibility that the event on E is already ready with data and as soon as E receives the message *m*2, it wants to pass the data to the task on node T, sending message *m*3 to T. If message *m*3 arrives on T before the message *m*1 from N, there is a problem, since task on T received a data on an input where it is not expecting one, since it was not yet informed about it by *m*1. Note that this does not depend on the order in which *m*1 and *m*2 are sent. The situation is shown in Fig. 6a. The problem can be solved by making node E responsible for sending *m*1 to node T, requiring it to do so before sending *m*3. This ensures that *m*1 and *m*3 are processed by T in the correct order, since messages are guaranteed not to overtake when they have the same sender and recipient. The situation is shown in Fig. 6b. However, adding a dependence is not the only scenario where multiple messages need to be properly synchronized. The other scenarios are more complex and while we tried to track all down and fix them, we failed in the end.

**Fig. 5:**
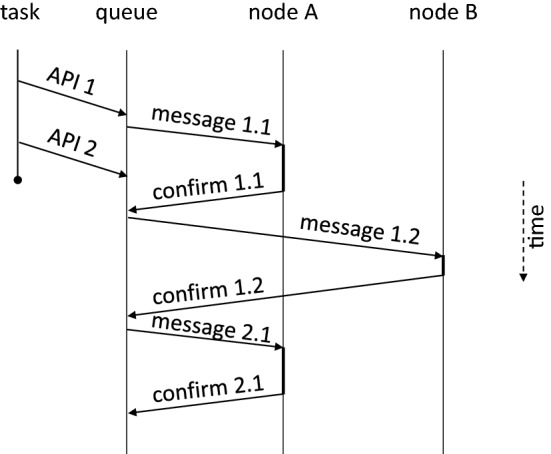
Visualization of the way that API calls made by a task are processed using a message queue and confirmation messages. The task makes two API calls. The first one is processed using two messages (1.1 to node A and 1.2 to node B) and the second one only needs one message (2.1 to node A). Note that the task finishes before the messages are fully processed

Clearly, a different approach was required. This came in the form of a message queue and message confirmations. The approach is somewhat similar to MPI_Ssend, which waits for the matching receive to be posted. However, we do not block the task that invoked the operation, making it more similar to MPI_Issend. But we go even further. All messages are processed in the background (like in the asynchronous MPI calls), not blocking the original task, but before a message can be sent out, all previous messages must be fully processed by their recipients (not just received like in MPI_Ssend). To facilitate this, all messages are put into a queue and after a remote node processes a message, it sends a confirmation to the node that sent the original message. After the confirmation is received, the next message can be sent out. An example is shown in Fig. 5. Another to view this solution is to see it as an asynchronously processed queue of remote procedure calls, where each call must complete before the next can start. Each OCR API called is translated to a sequence of these remote calls.

**Fig. 6:**
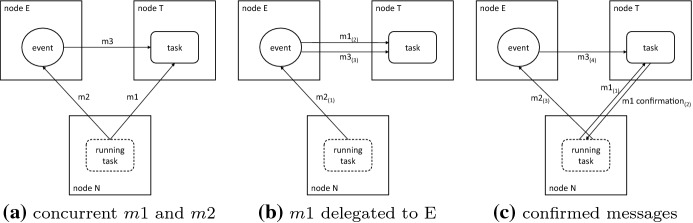
Distributed processing of messages adding a dependency from event on E to a task on T. The numbers in parenthesis indicate the guaranteed order in which the messages are sent (and also processed). No such ordering can be established in the first case between *m*1 and *m*3, since *m*1 is sent before *m*3, but *m*3 may be received and processed before *m*1, leading to a race condition

Going back to the example of adding a dependency (shown in Fig. 6), message *m*1 would be sent first and message *m*2 would be placed to a queue on node N. After *m*1 is processed by node T, it sends a confirmation message to node N, which then sends out the next message in the queue, which is *m*2. When processing of *m*2 triggers *m*3, we know that *m*1 has already been processed and the task on T is ready to accept the data. The situation is shown in Fig. 6c. Note that *m*2 and *m*3 will be confirmed as well. This ensures that if other operations are issued on nodes N and E, they are also processed in the correct order. The message queue is processed asynchronously in the background, which enables node N to continue executing its task, while it is still waiting for *m*1 to be confirmed. This is the aforementioned deferred execution model, where the runtime is not forced to apply the effects of all API calls immediately. We can see here that it is important in order to enable correct execution and prevent tasks from stalling.

This solution worked well, eliminating the race conditions encountered by the previous version of OCR-Vdm. However, a detailed study of the OCR specification revealed that it does not actually provide a solid foundation for this solution. While it made some obvious assumptions about how the OCR API calls are processed, which the deferred execution model and confirmation messages do not violate, there was no firm backing to confirm that it is a correct approach. This was the original impulse for extending the OCR memory model, as described in Sect. 3.1.

#### OpenCL support

The OpenCL programming model bears certain similarities to OCR. OpenCL buffers are similar to OCR data blocks, since any memory accessed by an OpenCL kernel has to come in a buffer, just like memory used by an OCR task comes in data blocks. One execution of a kernel on an NDRange corresponds to an OCR task. An OCR task is serial C/C++ code, but the kernel execution breaks down into many parallel work items. However, these work items are scheduled by the underlying OpenCL hardware and its driver, so it behaves as one object as far as the OpenCL command queue is concerned. The queue also defines synchronization using barriers and events, which is also somewhat similar to OCR events and dependencies.

We have extended OCR-Vdm with OpenCL support, allowing the creation of OpenCL tasks, which represented kernel executions. Data blocks could be passed to these tasks and they would be passed to the kernel execution as buffers. The implementation was limited to homogeneous clusters, where every node had the same OpenCL devices.

As systems like StarPU [3] demonstrate, it is possible to combine CPU and GPU tasks, achieving very good performance. However, the OCR would not provide a significant advantage over existing systems and we have decided not to try and replicate their work in OCR, with the best-case scenario being us matching their performance. We have considered porting the PEPPHER high-level framework [7] for OCR, to have a side-by-side comparison with StarPU, but early results on a CPU-only version have shown that we are unlikely to significantly outperform StarPU. Instead, we focused on other, more promising areas, especially NUMA, abandoning further development of OpenCL in OCR.

#### Fault tolerance

One of the goals of OCR was to provide fault tolerance. Storing all application data in data blocks makes this significantly easier, because it means that the OCR runtime system is aware of all application data and it has direct control over it. The only exception is local variables of running tasks, but the runtime system is also in charge of all tasks, so it can prevent tasks from interfering with processes that facilitate fault tolerance, like making snapshots. This makes fully automatic redundancy and recovery possible.

The deferred execution model allows tasks to be executed speculatively. If a task only works on copies of data blocks and no changes to other runtime objects are applied (which can be done simply by halting the processing of messages generated by the task), the task can run uninterrupted to completion and the runtime can later decide if and when to make the changes visible. This way, the runtime system does not even need to stop all tasks while it is creating a snapshot.

However, the OCR-Vdm implementation never included such speculative execution. Instead, the whole runtime can be paused. In that case, no new tasks would be started. Once the running tasks finished and there were no outstanding (send but not yet confirmed) messages, the state of the whole runtime system (including data in data blocks) was stored to disk. We were able to run an actual OCR application with regular checkpoints, simulate a crash, start a new job, recover the state from the last checkpoint, and restart the computation. The application ran to completion, providing the correct result.

The state could be read from the disk in a fresh instance of the runtime system with the same number of processes as the original one. Partial recovery (replace an individual failed node) or recovery with a different number of processes was not supported. Partial recovery would require a significant redesign of the whole OCR-Vdm checkpointing architecture. Changing the number of processes would be fairly easy to implement.

### OCR-Vsm

In the XSOCR implementation, the shared memory variant is basically a special case of the distributed memory variant. We have decided not to do it this way. The main reason was that OCR-Vsm was designed in a way that saved time and resources on a shared memory system, but could not be applied in a distributed memory environment.

In OCR-Vsm, the GUIDs are in fact pointers to the structures that they represent, eliminating the need for some kind of object lookup table. However, pointers would not be guaranteed to be unique in a distributed environment and we also need a way to find the objects, since every object was only stored on a single node. Also, the way the OCR-Vsm runtime synchronized access to shared objects would not translate well to a distributed environment. Finally, managing the state of data blocks can be streamlined in a shared-memory environment, since there is no need to support remote copies. Combined, we have observed as much as 1.62x speedup compared to OCR-Vdm running on the same hardware [24]. However, the XSOCR performance suggests that there is a significant space for improvement and it would be possible (with significant effort) to get the OCR-Vdm performance much closer to OCR-Vsm.

#### NUMA support

Since most modern high-performance CPUs come with an on-chip memory controller, non-uniform memory architecture (NUMA) is almost inevitable in a multi-socket system since more than one memory controller may be involved in handling a memory request [35]. Some processors go even further and may use NUMA even with a single socket (Intel KNL Xeon Phi can be configured with one, two, or four NUMA nodes; first-generation AMD EPYC processors have four NUMA nodes).

There is a significant performance penalty when a CPU core access memory that belongs to a different NUMA node. The actual performance impact depends heavily on the application, ranging from negligible to over 2x slowdown (as observed in both our and independent experiments [19, 34]). Most operating systems employ heuristics to place (and possibly also move) application data in memory in a way that improves locality. These approaches often work well for parallel applications based on multithreading, since threads rarely migrate between NUMA nodes and often already try to leverage memory locality to improve cache utilization.

In a task-based runtime system with a task-stealing scheduler, there is a significant chance of a task moving to a different thread from where it was created. This also means it probably moves to a different CPU core and possibly to a different NUMA node, reducing memory locality. Preventing tasks from migrating between NUMA nodes could lead to a significant imbalance. For example, in OCR, the whole computation starts with a single task. This task is responsible for creating other tasks, which in turn create more tasks and so on. If no task can migrate from the NUMA node of its creator, all tasks would stay in just one NUMA node.

We have started looking at NUMA in OCR when experimenting on the Intel Knights Landing architecture [19], which provides on-chip high-bandwidth memory and optional NUMA modes. We were only running the applications on one machine, using shared memory. But we have discovered that we get the best results by treating each NUMA node as a separate machine and presenting the hardware to the application as a distributed system with multiple compute nodes. OCR applications can use the OCR API to identify basic properties of the execution environment, like the number of compute nodes. Distributed applications running on a distributed OCR implementation (like OCR-Vdm) use this to determine data and work distribution and provide appropriate hints to the runtime system. Originally, OCR-Vsm always reported only one node. The NUMA-aware OCR-Vsm presents each NUMA node as a separate compute node. When the application provides a hint to place a data block or a task to one of these nodes, the data block is allocated in the memory of that NUMA node and the task is executed on a core that belongs to that NUMA node.

This is especially useful for applications that use the OCR way of exchanging data, by making them available in data blocks, rather than sending and receiving the data using additional memory buffers. This way, we do not get the extra overhead of memory copying as we would in an MPI application that would use send/receive to communicate among multiple processes running on the same machine.

In OCR-Vsm, we use shared memory, so all data can be accessed directly. We only instruct the application to configure itself as if it was running on a distributed system with multiple worker nodes. Then, we map the virtual worker nodes to NUMA nodes and when the application instructs the runtime to place a task or a data block to a certain worker, we restrict the scheduler (for tasks) or the operating system (for data blocks) to respect the required placement. We can still allow tasks to migrate between NUMA nodes if there is a significant imbalance.

#### Automatic task and data placement

While all of the existing OCR applications use hints to specify task and data block placement, it would be ideal if this could be done automatically. In a distributed environment, the penalty for a wrong decision is high, since we need to move data blocks between machines. In a shared-memory environment, we only pay the extra price of accessing memory from a non-local NUMA node, which is much smaller and works on a cache-line granularity, not whole data blocks. We have created an offline profile analyzer that automatically generates hints based on profiling data from a previous execution of the application [20]. The early results are promising, when dealing with regular, iterative applications. We have also started looking into the same problem in cases where there are multiple cooperating OCR applications running on a NUMA system concurrently [22].

### OCR-V1

The XSOCR team had plans for a “nanny” mode, where the runtime tries to check that the API calls made by the application are correct. During normal execution, the OCR runtime system makes very strong assumptions about the calls being correct, making debugging of the applications difficult. For example, when the API is called to establish a dependency between two objects, the application must provide two GUIDs. The first GUID must identify a valid event or data block and the second one must identify a valid event or task. The compiler cannot enforce these constraints, since all GUIDs have the same type in C.

Since we already had two different implementations, we have decided it would be the easiest to add another one, with application debugging in mind. OCR-V1 is a single-threaded implementation of the OCR specification. At runtime, it tries to catch as many bugs as possible. This includes ensuring that no object is used after it was destroyed, correct object types are passed to all API calls, etc. It also makes shadow copies of read-only data blocks to check that they have not been modified. This, combined with only having a single thread, makes it much easier to debug an OCR application during development. If the application does not work correctly in OCR-V1, it is most likely incorrect and the error is usually very easy to find with a debugger when OCR-V1 is used.

Obviously, OCR-V1 cannot find all race conditions, since it is not running tasks in parallel and it only uses a fixed task schedule, repeating the exact same order of tasks in repeated executions. To catch some of the race conditions that cannot be revealed this way, OCR-V1 can export a detailed trace of the API calls made by the application. We have created a trace analyzer, which looks at the trace and tries to find potential race conditions. The extended memory model that we created for OCR requires certain operations to happen in a specified order. For example, no operation on an object may come before its creation and no operation may come after it is destroyed. The memory model also defines the *happens-before* relation that defines which pairs of operations are guaranteed to happen in order, based on the way the application is synchronized. OCR-V1 exports sufficient data to allow the trace analyzer to reconstruct the happens-before relation among operations made by the application. By comparing the memory model rules (what has to be synchronized) and the reconstructed happens-before (what is synchronized), we can find various race conditions.

Even this determination is not definitive, since an application may also use data in data blocks to synchronize. For example, imagine a data block that contains a flag indicating whether a certain operation has already been performed. A task may look at the flag and if it is true, perform another operation. While there may be no happens-before relation between the two operations, they are in fact guaranteed to happen in order. They are just not synchronized using OCR events and dependencies. This may lead to false positives. There are also scenarios that can lead to false negatives.

Even when combined with the trace analyzer, OCR-V1 is not perfect. But it is still capable of discovering hard-to-debug race conditions and other errors in an OCR application. The trace analyzer is also a nice consequence of having a proper memory model.

## Experimental evaluation

The OCR is a research project. This is also reflected in the available applications, which are benchmarks or proxy applications that demonstrate how a certain type of application could be mapped to OCR. They focus on the structure of the computation, rather than the application-specific implementation details, like accurate physics simulation.

In general, the biggest obstacle to writing OCR applications is the requirement to store all data in data blocks and (even more so) the need to determine all data blocks needed by a task before the task starts. When porting an existing code to OCR, it is either necessary to significantly reorganize the code or introduce some mechanism that can split execution of a function into multiple consecutive tasks while preserving the correct state (like local variables). When the application needs a data block that has not been requested by the current task, the mechanism is used to create a new task that also requests the missing data block, but resumes the computation in the same state as before. Both code reorganization and splitting of functions have proven to be difficult.

A considerable effort (by the XSOCR team) was also spent on developing mpilite, an implementation of a subset of the MPI standard on top of OCR. However, mpilite used a “legacy mode” of XSOCR, which circumvented the requirement that all work is done by non-blocking tasks and allowed tasks to acquire data blocks during execution or wait for other tasks to finish. This mode is not supported by OCR-Vx.

The XSOCR team have implemented some well-known algorithms in OCR, like FFT, N-Queens problem solver, Cholesky factorization, Fibonacci numbers, BFS (Graph500 [39]), and 2D stencil. They have also ported existing codes of CoMD [40], miniAMR [45], HPCG [27], NEKBONE [26], and Tempest [44].

In the following, we provide performance results for four of our benchmark applications on different systems. As these experiments have been performed over a period of several years, some of them have been run on systems that have not been available for other benchmarks. All performance results reported are usually averaged over at least ten runs.

### Seismic

Our main contribution to the suite of OCR applications is the Seismic code. Seismic is a simulation of seismic wave propagation in a 2D environment. It is a port of the Seismic example code which is distributed with Intel Threading Building Blocks [31]. We have reimplemented the code in OCR. There are multiple variants of our OCR code, which were developed to test different ways of structuring the code and to test different experimental features (for example the channel events). Seismic is an iterative 2D stencil code, but we have made one significant change that makes it substantially different from the original TBB code. The TBB-based Seismic uses two consecutive parallel for loops for each iteration. This means that there are two (implicit) barrier operations in each iteration. We have eliminated these barriers and use a fine-grained synchronization, which allows the computation in consecutive iterations to partially overlap. Note that the Seismic code is included in the OCR-Vx distribution available at https://www.univie.ac.at/ocr-vx/.

While the details are beyond the scope of this article, a basic idea can be demonstrated using the channel event example depicted earlier in Fig. 4. Each column of workers represents one iteration and each row represents workers that process the same part of the data set. If we assume that the channel events are the only dependencies, then we can see that although task work(1,1) needs to wait for tasks work(0,0) and work(0,1) from the previous iteration, it does not have to wait for task work(0,3) to complete. Therefore, the task work(1,1) from the second iteration can run in parallel with task work(0,3) from the first iteration. While this organization could also be achieved using TBB, using the parallel for algorithm is a more natural solution in TBB. On the other hand, the fine-grained synchronization is very natural for OCR, especially with channel events. That being said, the programmer effort required to write the code in OCR is much higher than in TBB (with parallel for) and it would be possible to implement similar fined-grained synchronization in TBB with a similar effort.

An overview of the performance results obtained with the Seismic code is shown in Table 1. The table summarizes the performance of different implementations of the Seismic code on different architectures. Certain combinations of software and hardware are not available as the experiments were not performed at the time. For the new results (last column), we could not perform the experiment with XSOCR as the repository is no longer available. Since the original motivation for developing our OCR implementation was to use it on the Xeon Phi coprocessors, a significant part of the early experiments focused on this architecture [24]. There, we could see that our implementation matches or outperforms the OpenMP version of Seismic, which we created from the original TBB implementation by replacing the TBB parallel for loops with OpenMP parallel for loops. This is a very good result, since Seismic is the kind of code that OpenMP was designed to parallelize. A more detailed discussion of the experimental results for share-memory systems, NUMA, and distributed-memory follows in the respective sub-sections.

**Table 1:** Performance of different Seismic implementations on different architectures shown as the average execution time in seconds. The architectures are as follows: a dual socket server with 8 core CPUs (2x8), the Xeon Phi coprocessor (KNC), the Xeon Phi processor with NUMA disabled (KNL), the Xeon Phi processor with 4 NUMA nodes (KNL SNC4), and four socket server with 20 core CPUs (4x20). The data are a compilation of older experiments [19, 24] and new results. It shows that performance of OCR is competitive when compared with OpenMP and TBB. Also, it highlights the importance of NUMA-awareness on the NUMA systems (KNL SNC4 and 4x20)

	2x8	KNC	KNL	KNL SNC4	4x20
OpenMP	93.75	35.34	20.69	80.94	16.16
TBB	–	–	17.32	78.80	17.01
OCR-Vdm 1 node	117.57	–	–	–	29.44
OCR-Vsm NUMA-oblivious	72.50	35.53	20.93	34.04	5.10
OCR-Vsm NUMA-aware	–	–	20.76	19.53	4.67
XSOCR	–	–	20.68	20.66	–

#### Shared-memory systems

As can be seen from Table 1, our OCR implementation could match the performance of the OpenMP implementation on the Xeon Phi (with the setup used in the original paper [24], OpenMP took 35.34 sec and OCR-Vsm took 35.53 sec). The OCR version had the advantage of fine-grained synchronization, which is beneficial given the high number of threads on the coprocessor (60 cores, with 4-way hyperthreading, 240 threads in total). Even though OpenMP supports the nowait modifier which eliminates the barrier at the end of the for loop, the barrier had to be left in, since there are dependencies between the for loops that would not be preserved in that case.

However, the high number of threads was also the main reason why our implementation did not outperform the OpenMP code. With such a high number of threads, the TBB task scheduler takes a long time to start up and begin processing tasks on all 240 threads. In some configurations, the Seismic code could finish multiple iterations before all 240 threads started working. Also, while the OCR-Vsm runtime is highly de-centralized (there are no management threads and no “master lock”), there is still some overhead associated with such scale.

On the host (i.e., a normal server with two 8-core Xeon processors), OCR Seismic significantly outperformed OpenMP Seismic (72.50 and 93.75 sec, respectively). This is partly due to the fine-grained synchronization, but as our later experiments also revealed, due to better behavior with regards to NUMA.

#### NUMA

We have further explored the OCR performance on the Intel Xeon Phi architecture [19], moving from the coprocessors (KNC architecture) to the “bootable” CPUs (KNL). An interesting feature of these CPUs is the optional NUMA architecture. The CPU can be exposed as having 1, 2, or 4 NUMA nodes. Unlike NUMA interleaving on a traditional multi-socket server, where the memory architecture is the same and it is only exposed to the software in a different way, on the KNL, the memory management subsystem of the processor itself is reconfigured. NUMA-oblivious variants of Seismic (TBB, OpenMP, but also OCR-Vsm with NUMA support disabled) provided very good performance when the Xeon Phi CPU was run in the non-NUMA mode. The performance of all variants was comparable. There were different winners depending on the way the on-chip high-bandwidth memory was configured (with OpenMP and TBB among the fastest, closely followed by different OCR implementations). But when the CPU was switched to the mode with 4 NUMA nodes, the situation changed significantly, with NUMA-aware OCR-Vsm being the clear winner, followed by XSOCR. We have discovered that exposing the 4 NUMA nodes to the OCR the same way as we would expose a compute cluster with 4 nodes provides significant performance benefits. Since most OCR applications are written for distributed execution, they do not a have problem adapting to multiple nodes, whether they are distinct machines or just different NUMA nodes. Favoring local computation and minimizing data movement is beneficial in both cases.

While experimenting with the KNL, we have also tested the application on a traditional multi-socket NUMA server. The results suggested that in that case, the difference is even more pronounced, but as they were not the focus of the paper, we did not report the results. But they showed that NUMA was a highly relevant topic for runtime systems like OCR. To provide up-to-date data, we have now re-run these experiments on a modern NUMA system. The machine contains four Intel Xeon Scalable Gold 6138 processors (Skylake architecture, 20 cores, AVX-512 support, 32 KB L1 data cache per core, 1 MB L2 cache per core, and 27.5 MB last-level cache in total). There are 24 memory modules installed in the machine to fully utilize the 6 memory channels per CPU. Each module has an 8 GB capacity. The total number of physical cores is 80. There is one NUMA node for each processor, meaning the server has a total of four NUMA nodes, each with 20 cores. The OpenMP Seismic needs 16.16 sec to run the test workload, the pure TBB version needs 17.01 sec, and the NUMA-aware OCR-Vsm takes 4.67 sec to complete.

Again, it is important to keep in mind that the TBB and OpenMP Seismic codes are NUMA-oblivious and their performance could be significantly improved, probably to (at least) match the OCR-Vx performance. Dealing with NUMA systems in OpenMP is already well studied. TBB allows the creation of multiple independent thread pools, which can be bound to cores belonging to specific NUMA nodes, so it is also possible to control where TBB tasks are executed. But the programmer effort would be significantly increased, especially in the case of TBB, possibly matching the complexity of the OCR code.

#### Distributed-memory systems

The OCR-Vdm also showed some promising results. On an 8-node cluster, it could reach a 7.27x speedup over a single node when running Seismic, assuming that the task and data block affinity hints were set up correctly. In some other applications, like the XSOCR-developed Stencil2D, the performance was not as good. Compared to the XSOCR runtime, OCR-Vdm had a higher per-task overhead and Stencil2D uses a large number of helper tasks, making any such overhead much more pronounced. As a point of comparison, according to the XSOCR team’s results [10, 39, 41], the XSOCR running Stencil2D can keep up with MPI until 64 nodes. It is somewhat slower (reaching about 80% of the throughput achieved by MPI), but exhibits similar weak scaling. The scaling of XSOCR drops at 256 nodes, but it still achieves over 60% of MPI. The XSOCR Stencil2D experiments also use hints to define task and data block placement.

In Table 1, you can see that the performance of OCR-Vdm is quite poor on the new many-core server compared to other Seismic implementation. This is simply due to the poor scaling of the OCR-Vdm implementation to such a high number of cores. In fact, the performance could be slightly improved by artificially reducing the number of tasks so that not all cores would be utilized.

The main conclusion to be drawn from our experiments is that properly managing NUMA is critical for task-based runtime systems and that a lot of inspiration can be drawn from distributed runtime systems. Given the right circumstances, the OCR can provide performance that is comparable to the alternative solutions, but for NUMA and distributed systems, the application developer needs to provide hints that provide good placement of tasks and data blocks. We have developed a system that automatically determines hints in iterative applications based on profiling with some success [21] and the XSOCR team have been investigating distributed schedulers that could work without the hints, but neither has yet provided a way to automatically determine good task placement in the general case [10].

### Stencil2d

The *stencil2d* application was obtained from the now defunct application repository that accompanied the OCR implementation created by Intel and Rice University [38]. The stencil2d application is a proxy application that applies a stencil operation to a square grid. It is possible to specify the size of the grid, the number of iterations, and the number of rectangular sub-grids that the whole grid is split into (each sub-grid is a data block). The sub-grids are then processed in parallel, so there is one strand of tasks for each sub-grid. The application prints out the number of MFLOPS it achieved. We present performance results on two different shared-memory systems, an Intel machine with a total of 80 cores (four Intel Xeon Gold 6138 20-core CPUs) and an AMD system with a total of 64 cores (two AMD Epyc 7501CPUs). We compare our OCR-Vsm versions (both NUMA-aware and NUMA-oblivious) to a TBB version. The stencil application is always configured to use as many sub-grids as there are cores (80 on Intel, 64 on AMD). The data size is $10240 \times 10240$. The code performs 1000 iterations.

As shown in Table 2, the OCR-Vsm variants are much faster (more than 2x) than the TBB version. As expected, on both machines, the best performance is obtained by using OCR-Vsm with the NUMA-aware scheduler.

**Table 2:** Performance of the stencil2d benchmark in terms of speedup over TBB on two different machines

Machine	Code version	MFLOPS	Speedup over TBB
Intel	TBB	47403	1.00
	OCR-Vsm NUMA-oblivious	127266	2.69
	OCR-Vsm NUMA-aware	134148	2.83
AMD	TBB	50758	1.00
	OCR-Vsm NUMA-oblivious	116424	2.29
	OCR-Vsm NUMA-aware	118204	2.33

### Face detection

Another application that has been implemented with OCR-Vx is a real-world face detection application we original developed in the PEPPHER project [7] with a high-level pattern-based programming framework [5] on top of the StarPU runtime system. The application utilizes routines from the open-source computer vision library OpenCV [28], which have been slightly reengineered to conform to the tasking model. The application is implemented in accordance to a pipeline pattern with 5 pipeline stages. The first stage reads an image from a file, the second stage converts the image to grayscale, the third stage does the actual detection of faces, the fourth stage marks the detected faces with squares, and the final stage writes the resulting image with detected faces marked to an output file. For the measurements, the face detection application processes a set of 500 images of 360p resolution, each containing an arbitrary number of faces. We present speedup measurements on a machine with 16 cores (2 Intel Xeon E5-2650 CPUs, 2.0 GHz, 128GB RAM). We compare our OCR-Vsm (non NUMA-aware) variant to a StarPU variant, a TBB version that uses the pipeline algorithm of TBB, and to a sequential C++ version, which is used as a baseline. We have used GCC 5.3.0 compiler, OpenCV version 3.3.1, StarPU 1.2.4, TBB 4.2, and our own OCR-Vsm implementation.

**Table 3:** Performance of different version of the face detection application on a 16 core machine (2 Intel Xeon E5-2650 CPUs)

Code version	Execution time (secs)	Speedup over sequential C++
sequential C++	159.56	1.00
OCR-Vsm	11.24	14.19
TBB	11.40	13.99
StarPU	17.47	9.13

Table 3 shows the execution times of the different code versions and speedup numbers of the parallel versions with respect to the sequential C++ variant. The OCR-Vsm version achieves a speedup of more than 14 using all the 16 cores in the machine, very closely followed by the TBB version. The StarPU version achieves a lower speedup of 9.13. We consider this a very good result for our OCR-Vsm implementation, given the fact that the TBB pipeline algorithm is highly optimized for shared memory systems. Of course, given the low-level of the OCR API, the TBB version is more compact in terms of overall lines of codes and also less complex.

### Levenshtein on a heterogeneous system

We have also evaluated our OpenCL extension of OCR on a 16 core system (2 Xeon E5-2650 CPUs) equipped with an NVIDIA K20m GPU. The performance results are summarized in Table 4. Using a simpler application (Levenshtein edit distance), we could observe a significant speedup when using GPU tasks for the edit distance computation compared to the CPU-only variant (down from 70.51 to 22.68 sec). However, as the data transfers were not optimized by the runtime system, it was clear that the performance could still be significantly improved. This also impacted the performance of a hybrid GPU+CPU execution, where we performed a static distribution of tasks between the CPU and the GPU. This hybrid version was actually slower (28.65 sec) than the GPU-only version. This experiment clearly showed that it is not easy to balance execution between CPU and GPU, especially since we were using a static work distribution.

**Table 4:** Performance of Levenshtein benchmark with OpenCL extension of OCR on a CPU/GPU system (2 Intel Xeon E5-2650 CPUs, 1 NVIDIA K20m GPU)

Machine configuration	Execution time (secs)	Speedup over CPU only
CPU (2 × 8 cores)	70.51	1.00
GPU (K20m)	22.68	3.11
CPU + GPU	28.65	2.46

Since in our OpenCL extension to OCR, OpenCL tasks are separated from normal OCR tasks, the runtime cannot redistribute them automatically, without some kind of task variant support. So, while this demonstrated that GPU tasks can be integrated into OCR, it was apparent that significant improvements would have to be made to the task scheduler to make it a viable alternative to systems like StarPU, which support heterogeneous systems via unified offloadable task abstractions called codelets.

## Related work

The task-based design of OCR is similar to other task-based runtime systems like StarPU [3], TBB [31], OMPSs [11], or HPX [29]. However, while those other runtimes have been primarily designed for shared-memory systems, OCR has been targeting distributed-memory systems from the outset. Support for distributed-memory architectures is inevitable for addressing large-scale parallel systems and it is facilitated in OCR through the concept of data blocks, which decouple data from its representation in the memory system, giving the runtime system greater control over data placement and communication optimization.

As explained earlier, our work has been significantly inspired by the Intel Threading Building Blocks (TBB, [31]) library. Some versions of OCR-Vx also use the TBB task scheduler and memory allocator for their implementation. The NUMA-aware task scheduler in our shared-memory implementation OCR-Vsm was also to some degree inspired by the TBB task scheduler (the TBB source code is available as open-source). Another important source of inspiration was of course the XSOCR implementation (also open source, although the public repository is no longer available) [10]. The sources for P-OCR [32] were not made public.

The TBB library is related to OCR in one other aspect. Even though TBB and OCR represent task dependencies in a very different way, there is a fundamental similarity between the two. By default, there is no persistent representation of the result of task execution. Many of the other task-based runtime systems (discussed later in this section) have events or futures that can be used to query the task result and build further dependencies, irrespective of whether the task itself has finished. In TBB, once a task finishes, there is no way to use it as a source of a dependency. As a result, dynamically building the task graph during application execution requires careful planning or synchronization, to ensure that the tasks are guaranteed to be in a valid state. By default, each OCR task comes with an associated event that represents the result of the task. This event also does not persist after the task finishes, raising the same concerns as TBB. However, there are different types of events that are not destroyed automatically, but using these requires extra programmer effort.

UPC++ [47], Charm++ [30], C++ concurrency extensions proposal [1], or Dask [42] use (persistent) futures. OmpSs and the underlying Nanos++ runtime system [11], OpenMP 4.0 tasks, and StarPU [2, 3] can use data dependencies to build the task graph. X10 [43], Habanero-Java [12], Chapel [14], and Cilk [33] use the spawn-sync model, where (implicit or explicit) sync operation is used to wait for all tasks that have been created earlier to finish. Many of the other systems (e.g., OpenMP tasks and TBB) also provide easy-to-use support for this model.

UPC++, Charm++, X10, Habanero and Chapel are also good examples of alternative distributed task-based runtime systems. But there are significant differences in how they approach the problem. UPC++ is based on the partitioned global address space (PGAS) model, whereas OCR uses relocatable data blocks. Instead of tasks, Charm++ uses “chares” to express parallel computation. Chares are objects which can communicate via messages with each other and possibly execute a long sequence of interleaved computation and communication. Legion [6] is a data-centric runtime system, where the runtime system is responsible for managing the distributed storage of the data (including data movement), based on information about the data structured provided by the application. The data structure is also the basis for defining and managing parallel execution. In OCR, the location of data blocks can be used as one of the inputs for the scheduling algorithm, but the existing implementations focus much more on the tasks themselves. Unlike Legion, if the data in an OCR application are split into many smaller data blocks, the runtime system has no notion of these data blocks being related.

Some of the runtime systems can also support heterogeneous architectures like, for example, Legion and StarPU. The approach used by StarPU could to a great extent be used in OCR with our OpenCL extension. As stated earlier, we have not implemented this, since we could not see a way to not only replicate their work, but also further improve upon the state-of-the-art.

The OCR-V1 is not unique in being single-threaded. Many of the other runtime systems can be configured to use only one thread. Similarly, having a more strict debug mode is a common practice. However, XSOCR never actually implemented the “nanny mode,” so OCR-V1 is the only such OCR implementation. There is a similar tool to our OCR-V1 trace analyzer. GT-Race [47] is based on XSOCR and focuses on race conditions in data block accesses, which our trace analyzer does not deal with.

One idea behind early OCR development was to create a universal runtime system [33] that could be used as a basis to implement other runtime systems and high-level programming tools. This has never materialized. As we have just discussed, there are many different ways to design a distributed runtime system and the OCR design was not sufficiently universal to easily accommodate all of them. Also, making the runtime system responsible for all non-transient data (as data blocks) and all computation (only OCR tasks are allowed, no main thread or user threads) made it difficult to map the other programming models and runtime systems that give the programmer more control. Recall that to provide an MPI-like programming model, mpilite relied on “legacy code” extensions of OCR. Or, to give another example, TBB is explicitly designed to be able to closely coexist with other threading models and libraries, making the OCR’s own-all approach incompatible.

## Conclusion

In this article, we have provided the big picture of the OCR ecosystem. As the creators of OCR-Vx, we mostly presented our point of view, but thanks to close cooperation with the XSOCR team, we could also provide their perspective where appropriate. While the merits of different design decisions made in OCR are open for debate, the benefits and the importance of the stand-alone OCR specification cannot be overstated. Even for an early-stage research project, spending the resources to come with such a document has provided significant advantages down the line. Having two separate implementations is rather unique and would most likely not be possible without the specification. As demonstrated by Internet Standards [9], having independent implementations greatly helps in creating good specifications.

From the adopters’ point of view, the biggest hurdle to overcome are the OCR data blocks. However, the decoupling of data from storage will be even more important for future architectures where we will see even more complex memory hierarchies that may change dynamically or where the runtime system could configure the memory hierarchy based on application requirements (e.g. non-uniform cache architectures and dynamic cache/scratch-pad architectures). Furthermore, the fact that data movement is the most expensive operation (in terms of performance and energy) mandates flexible techniques for managing application data.

Having to store data in these data blocks is not too convenient, but the main problem for many application developers is the fact that a task needs to request all data blocks before it starts and it is not possible to acquire further data blocks while the task is running. This design decision is very important for OCR and cannot be easily removed, since it is necessary to allow OCR tasks to be non-blocking. Once an OCR task starts, it is able to run to completion without waiting for any other task. This is very useful for the runtime systems, allowing interesting optimizations (like the deferred execution model), but the price for applications is significant. It is important to keep in mind that OCR is intended as a foundation for building higher-level programming models, which could hide much of this complexity from application developers.

As for the runtime systems and their performance, it was demonstrated that OCR can achieve good performance in both shared- and distributed-memory systems, but at the moment, a significant effort is required from the application developers to provide correct hints to the runtime system. As our experiments have shown, it is possible to take an application that was designed like this for a distributed OCR and use it to efficiently use a (shared-memory) NUMA system. We believe the same could be applied to other runtime systems.

We believe that OCR has introduced important concepts required to deal with the increased scale, complexity, and performance variability of future parallel architectures. Maybe the most important lesson learned from the OCR developments is that data and computation need to be treated at the same level, which is ensured by the concept of data blocks in OCR. Thus, unlike most other task-based runtime systems, OCR allows decoupling the data from memory, akin to the decoupling of computations from compute units as provided by tasks.

We believe that there are many opportunities for future research in task-based programming models, for example, to address the challenges coming with new architectures like FPGAs or PIM (processor in memory) architectures. Also, as OCR has been designed primarily as a low-level runtime on top of which higher-level programming models can be built, the level of abstraction provided to application programmers is rather low. This is a general problem shared by all of the current task-based runtime systems and is a major obstacle for a broad adoption of such systems for application development. Thus, it is important that high-level programming models and tools are developed on top of task-based runtimes. Also, interoperability of task-based runtimes is an important issue [4] given the trend toward application-specific architectures with their own specialized runtimes. All these issues present interesting opportunities for future research.

Finally, we believe that proper future standardization efforts could significantly facilitate wide-scale adoption of task-based runtimes.

## Footnotes

1 To create any task, it is first necessary to create a task template, which specifies the action performed by the task. A common practice is to create all templates when the application starts. Furthermore, an application would most likely create multiple data blocks to store application data, create some tasks to start the computation, and assign the data blocks to these tasks.

2 There were a few technical issues, mainly due to the fact that some enumerations in the header files were not assigned fixed numerical values. Combined with the fact that certain optional OCR features can be turned on and of using compile-time constants, special care had to be taken to ensure that the numerical values were the same in different library builds. This has been fixed in a later version of the headers.
